# Colostomy fistula caused by segmental absence of intestinal musculature: a case report

**DOI:** 10.1186/s40792-023-01701-z

**Published:** 2023-06-22

**Authors:** Jumpei Shibata, Kota Inagaki

**Affiliations:** 1 Department of Surgery, Nishichita General Hospital, 3-1-1 Nakanoike, Tokai, Aichi 477-8522 Japan; 2 Department of Surgery, Nishio Municipal Hospital, 6 Kamiawahara, Kumami-cho, Nishio, Aichi 441-8570 Japan

**Keywords:** Segmental absence of intestinal musculature, Colostomy, Fistula, Case report

## Abstract

**Background:**

Segmental Absence of Intestinal Musculature (SAIM) is a rare condition associated with intestinal obstruction and perforation. Colostomy fistula as a presentation of SAIM and their association with anastomotic failure have not been previously reported. This case report aimed to raise awareness of this unique manifestation and its potential implications.

**Case presentation:**

A 58-year-old male with a history of type 2 diabetes, hypertension, and lumbar hernia presented with diarrhea. Lower gastrointestinal endoscopy revealed a tumor in the rectum, for which he was diagnosed with a well-differentiated adenocarcinoma. The patient underwent a laparoscopic Hartmann operation. After the operation, an entero-entero-fistula was identified at the sigmoid colostomy site. Subsequently, laparoscopic reconstruction of the colostomy was performed, and the patient had a favorable postoperative course without complications. Histopathological examination confirmed the localized absence of the muscularis propria in the resected colon, with fibrosis and nearby ganglion cells.

**Conclusions:**

This case highlights the rarity of a colostomy fistula as a manifestation of SAIM and emphasizes the need to consider SAIM in the differential diagnosis for such cases. The presence of SAIM-affected lesions poses a risk of anastomotic failure, underscoring the importance of assessing the risk of complications during future surgeries. Surgeons should be aware of the etiology and potential implications of SAIM to ensure appropriate management and minimize postoperative morbidity. Further studies are warranted to explore the underlying mechanisms and optimize surgical strategies for patients with SAIM and its associated complications. Increased awareness among clinicians is crucial for timely diagnoses and tailored interventions to improve patient outcomes.

## Background

Segmental absence of the intestinal musculature (SAIM), a rare condition characterized by partial or complete defects in the intestinal muscularis propria, is associated with intestinal obstruction and perforation in all age groups [1]. Originally considered as a cause of intestinal obstruction and perforation in the newborns [2–4], SAIM has also been reported in adults [1, 5, 6]. Although the precise etiology of SAIM remains to be unknown, suspected causes include dysplasia of the intestinal muscle layer during embryonic development, ischemia and/or infarction, interruption of the blood supply, and trauma [7, 8].

Colostomy creation is a beneficial procedure employed to divert the fecal route in the management of various pathological conditions such as congenital anomalies, colon obstruction, inflammatory bowel disease, trauma, and gastrointestinal malignancy [9]. However, colostomy-related complications, such as stomal retraction, skin excoriation, and parastomal hernia, can occur [10]. Nevertheless, a colostomy fistula is a rare complication.

In this report, we present a case of a colostomy fistula resulting from SAIM. This report aimed to raise awareness about the potential risk factors associated with colostomy creation in patients with SAIM.

## Case presentation

A 58-year-old male presented with diarrhea. His medical history included type 2 diabetes, hypertension, and a lumbar hernia. He had a history of smoking and occasional alcohol consumption. Physical examination results were unremarkable. Initial laboratory tests revealed a serum hemoglobin level of 12.7 g/dL, a serum carcinoembryonic antigen level of 1.5 ng/dL (reference range: 0–2.5 ng/mL), and a carbohydrate antigen 19–9 level of 13.0 ng/dL (reference range: 0–37 U/mL). Lower gastrointestinal endoscopy revealed a half-circumferential rectal tumor. A biopsy confirmed a well-differentiated adenocarcinoma. Contrast-enhanced computed tomography (CT) revealed focal wall thickening of the rectum (T3) with several enlarged perienteric lymph nodes (N2) and extramural vascular invasion, without evidence of metastasis (M0). The patient received two courses of neoadjuvant chemotherapy of mFOLFOX6 regimen, followed by laparoscopic Hartmann's operation. Arterial flow at the edge of the sigmoid colostomy was preserved during surgery. The perienteric peritoneal metastasis was completely resected. Postoperative histopathological findings revealed a residual adenocarcinoma with a TNM score of ypT3N2aM1c. The patient was discharged from the hospital on postoperative day 13.

**Fig. 1:**
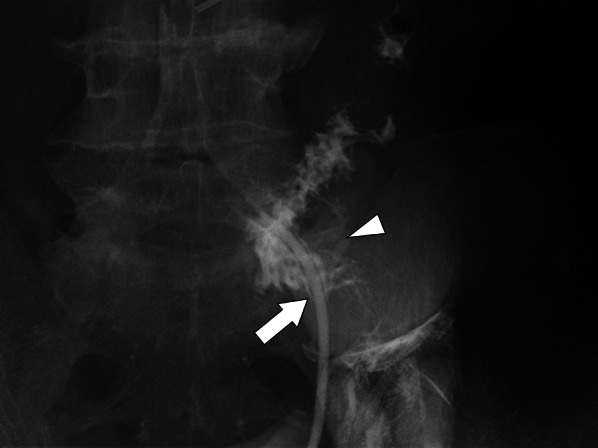
Fistulography from the orifice beneath the normal sigmoid stoma. Fistulography revealing an entero-entero fistula originating from the orifice beneath the normal sigmoid stoma. 8 Fr catheter inserted from the fistula (arrow) and arrowhead shows the stoma orifice

**Fig. 2:**
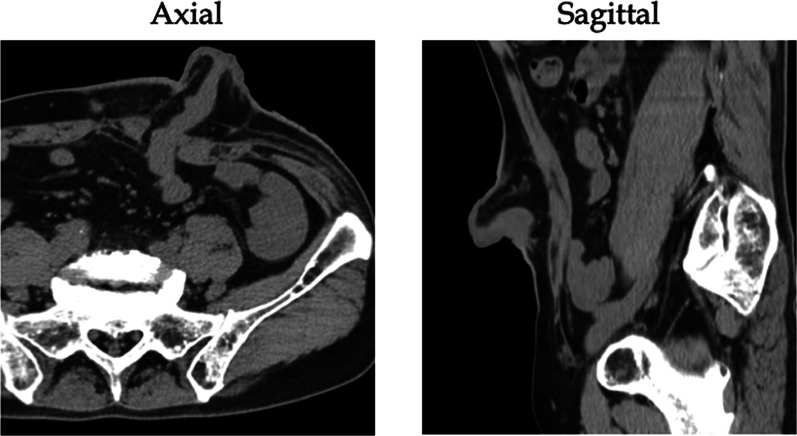
Abdominal computed tomography. Computed tomography of the abdomen showing no evidence of intra-abdominal abscess or inflammation around the stoma

**Fig. 3:**
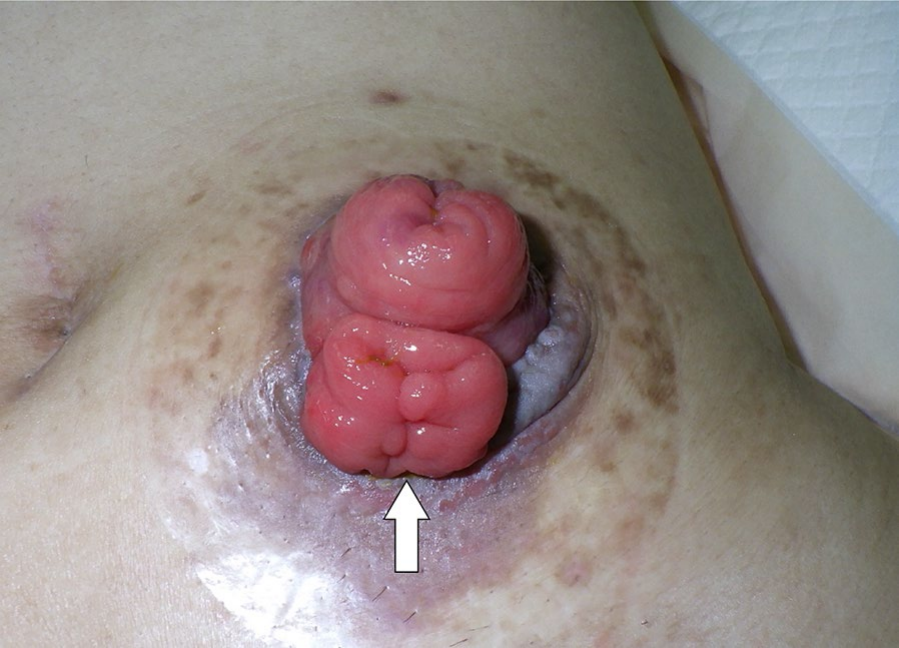
Enlarged entero-entero fistula before stoma reconstruction. Colonic mucosa prolapse through the fistula (arrow) leading to painful defecation. Although the original stoma had been single-barrel, it appeared like a double-barrel stoma

On postoperative day 28, the patient reported an abnormal orifice in the stoma and painful defecation. Physical examination revealed a colostomy fistula at the 6 o'clock position of the stoma orifice (Fig. 1). No intra-abdominal abscess or inflammation was observed (Fig. 2), and adjuvant chemotherapy with mFOLFOX6 was administered. However, the colostomy fistula gradually enlarged (Fig. 3) and painful defecation persisted. Consequently, laparoscopic colostomy reconstruction was performed.

**Fig. 4:**
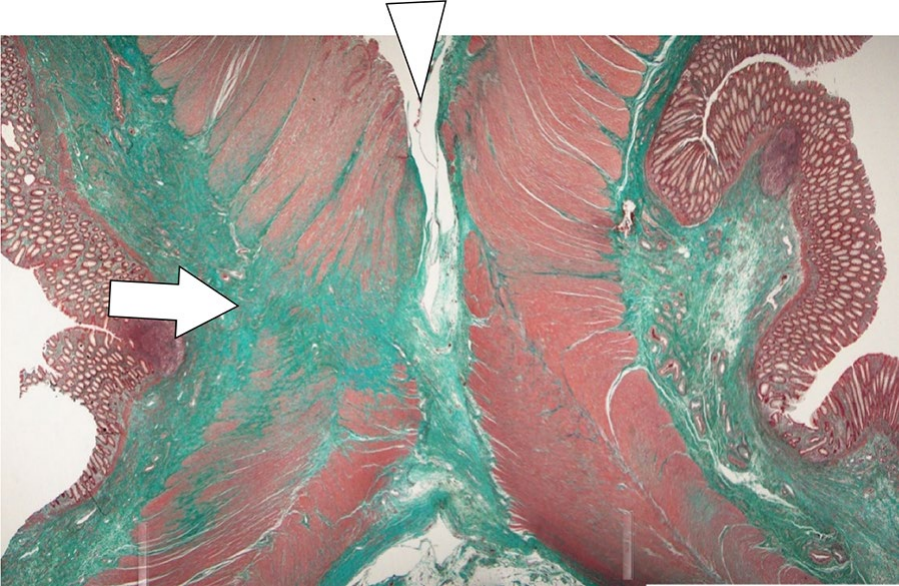
Gross findings of the resected fistula and normal stoma. Gross examination of the resected specimen showing the normal stomal orifice (arrowhead) and the fistula orifice (arrow). The fistula colon exhibits a loss of muscularis propria

**Fig. 5:**
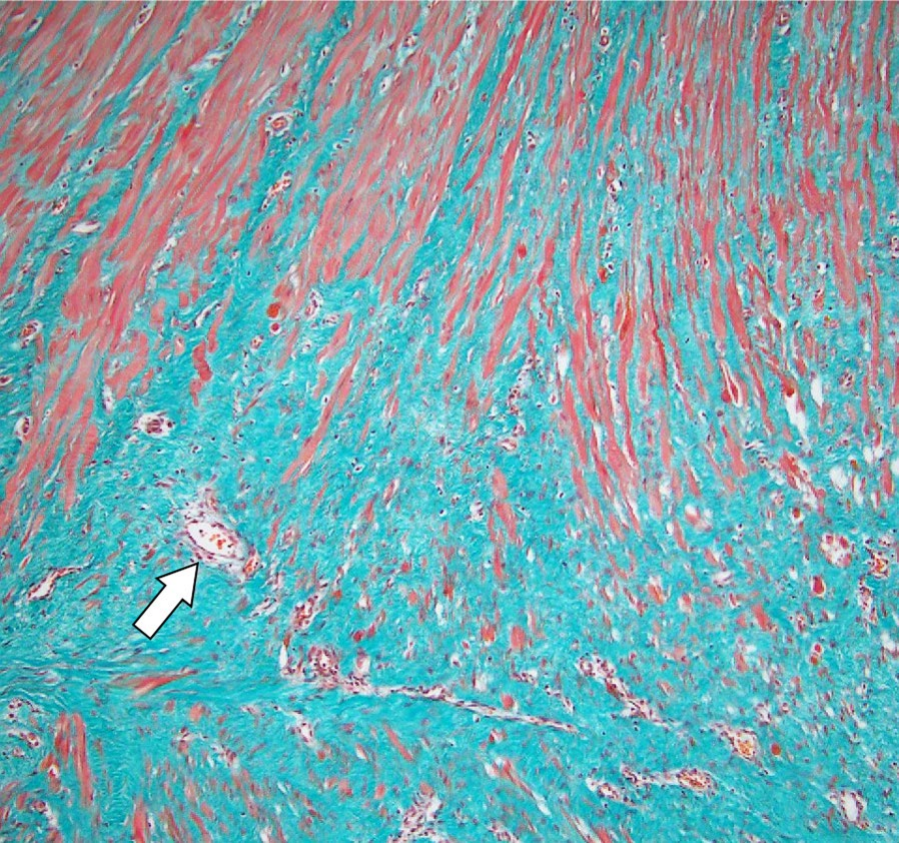
Pathological findings of the fistula. Masson trichrome stains of the fistula revealing fibrosis replacement (blue area) where muscularis propria (red area) have been replaced by fibrosis. Adjacent ganglion cells were observed (arrow)

During surgery, no necrosis or abscesses were observed in the abdominal cavity. The edge of the stoma was resected, and colostomy reconstruction was performed without complications. Macroscopic examination of the resected intestinal tract revealed thickening of the surrounding intestinal wall, without the evidence of a diverticulum (Fig. 4). Histopathological analysis of the resected colon with the fistula showed a fibrosis replacement where originally muscularis propria exists, with adjacent ganglion cells (Fig. 5). The patient did not experience any adverse events or gastrointestinal symptoms following surgery.

## Discussion

We present a case of colostomy fistula that developed after a laparoscopic Hartmann's operation. To the best of our knowledge, this is the first reported case of a colostomy fistula caused by SAIM. This case is instructive for two reasons. First, a colostomy fistula can be a presentation of SAIM in addition to intestinal perforation and stenosis. Second, SAIM-affected intestinal lesions can be multiple, and the presence of residual SAIM increases the risk of anastomosis failure. Therefore, it is crucial to assess the risk of complications when planning future surgeries for patients with SAIM.

SAIM is a rare condition associated with intestinal obstruction and perforation due to partial or complete defects in the intestinal muscularis propria [1]. The absence of the muscularis propria in the intestine predisposes it to wall thinning, which can lead to spontaneous focal perforation or stenosis of the intestinal tract [5]. Although SAIM is often diagnosed as an intestinal obstruction or perforation in newborns, cases have also been reported in adulthood [1, 5, 6]. The sigmoid colon is the most commonly affected organ (46%), followed by the ileum (19%), and jejunum (12%), with a mortality rate of approximately 30% after perforation [11]. In our case, colostomy was performed in the sigmoid colon, which is the most frequently affected area. SAIM cases are typically diagnosed after emergency surgery for intestinal stenosis or perforation [12, 13], but no previous cases of colostomies caused by SAIM have been reported.

However, the etiology of SAIM in adults remains unclear. In contrast, two etiologies have been recognized in newborn SAIM. The first is a focal congenital anomaly that occurs during intestinal development, and the second is intestinal ischemia that occurs postnatally or later in life or postnatally [7]. Ischemia leads to injury to both the mucosa and muscularis propria. However, these tissues have differing regenerative capacities [14], resulting in the regeneration of the mucosa without the muscularis layers. Histological findings in patients with prior ischemic changes revealed fibrosis with the Auerbach plexus remaining visible in the region of muscular absence [11]. Given that there were no concomitant congenital anomalies in our case and the presence of fibrosis and neural ganglion cells surrounding the affected area, we speculated that the loss of the muscularis propria was due to ischemia during early fetal life.

However, the mechanisms underlying SAIM symptoms are not fully understood. It is reasonable to assume that SAIM-affected intestinal segments are susceptible to increased internal intestinal pressure, which may contribute to the development of SAIM-associated diseases. Many patients diagnosed with SAIM-associated perforation have a history of conditions that increase intestinal pressure, such as carcinoma, trauma, intestinal obstruction, and surgery [11]. Although no previous studies have specifically assessed the stoma’s intraluminal pressure, we hypothesized that the intracolonic pressure at the stomatal orifice may be higher than that in the other parts of the intestine. Intestinal pressure is determined using Laplace’s law, which states that the tension on the wall of a cylinder is proportional to both the pressure of its contents and its radius [15]. The radius of the stoma orifice is smaller than that of the other parts of the intestine due to the presence of the mesentery and omental appendices. These anatomical features contributed to the smaller radius, resulting in increased pressure and fistula formation.

An internal fistula is an abnormal connection between one part of the intestine and another part of the body. Among these, the entero-entero-fistula refers to the connection between the different parts of the intestine. Enteric fistulas can be induced by foreign bodies, radiation, inflammation (e.g., Crohn's disease), infection (e.g., tuberculosis and actinomycosis), epithelialization, neoplasia, and distal obstruction [16]. Crohn's is the most common cause of spontaneous fistulas. However, our patient did not exhibit any other symptoms throughout the course that would suggest Crohn’s disease as the cause of the enteric fistula.

Patients with acquired SAIM may have a history of bowel symptoms or have undergone multiple surgical procedures. Identification of the acquired SAIM in the resected specimen should alert the surgeon to the possibility of additional areas lacking the muscularis propria in the remaining gastrointestinal tract [1]. This is particularly important for surgeons and patients considering the restoration of gastrointestinal continuity, because SAIM-affected lesions can increase the risk of anastomotic failure.

## Conclusions

Here, we report a case of colostomy fistula caused by SAIM following distal gastrectomy. An entero-entero-fistula can be an intestinal manifestation of SAIM, and surgeons should be aware of its etiology and the likelihood of additional areas of muscularis propria absence in the remaining gastrointestinal tract in patients with SAIM to plan future surgeries accordingly.

## Data Availability

All data supporting our findings are contained within manuscript.

## Abbreviations

SAIM: Segmental absence of intestinal musculature
CT: Computed tomography
